# 2349: Development of a Pediatric Hydrocephalus Severity Index to predict long-term clinical outcomes

**DOI:** 10.1017/cts.2017.264

**Published:** 2018-05-10

**Authors:** Rowland Han, Yan Yan, Abhaya Kulkarni, T.S. Park, Matthew Smyth, Jennifer Strahle, David Limbrick

## Abstract

OBJECTIVES/SPECIFIC AIMS: To create a composite index, referred to as the Pediatric Hydrocephalus Severity Index (PHSI), to classify the severity of disease at baseline and predict outcomes among children treated for hydrocephalus. METHODS/STUDY POPULATION: The Hydrocephalus Outcome Questionnaire will be administered in person or online to the parents of 150 patients between the ages of 5 and 18 years who are followed at the Neurosurgery Clinic at St. Louis Children’s Hospital for hydrocephalus. Patients must have been diagnosed and treated for hydrocephalus at least 6 months prior to the survey date. Potential participants are excluded if their health status changed during the 4 weeks prior to survey date, as determined by the child’s parents. Potential risk factors (see anticipated results) will be identified on retrospective medical record review. We will create a clinical prediction rule, called the PHSI, to stratify patients on likelihood of experiencing a poor long-term outcome after surgical treatment. Participants will be classified as “good” or “poor” outcome based on thresholds set for questionnaire results. We will use a combination of bivariate analysis and clinical reasoning to restrict the number of factors for further analysis, and multivariate logistic regression to build a predictive model for poor outcome. Creation of the PHSI will involve assigning integer values to adjusted odds ratios for significant risk factors at a 95% confidence level. RESULTS/ANTICIPATED RESULTS: Risk factors that we anticipate will be predictive of long-term clinical outcome include signs and symptoms at onset (bulging fontanel, splayed sutures, papilledema, up-gaze palsy, headache, vomiting, lethargy), head circumference above the 97th percentile, frontal-occipital horn ratio greater than 0.4, etiology of meningitis or neonatal intraventricular hemorrhage, central nervous system comorbidities (seizures, Chiari malformation, scoliosis, periventricular leukomalacia), preoperative infection or sepsis, and frequent shunt revisions or infections. We hypothesize that a PHSI will be a valuable tool for stratifying patients in future research studies, as well as aiding prognosis in clinical situations. DISCUSSION/SIGNIFICANCE OF IMPACT: A validated composite PHSI would be a major advance in clinical hydrocephalus research and practice. A PHSI would allow investigators to stratify patients based on initial presentation for clinical research studies, which may in turn lead to the establishment of more standardized treatment guidelines. It would also facilitate studies investigating differential utilization of healthcare resources based on disease severity. Clinically, a PHSI would better equip physicians to counsel parents on what to expect for their child or future healthcare resource requirements.

